# Design and Implementation of Opportunity Signal Perception Unit Based on Time-Frequency Representation and Convolutional Neural Network

**DOI:** 10.3390/s21237871

**Published:** 2021-11-26

**Authors:** Zhongliang Deng, Hang Qi, Yanxu Liu, Enwen Hu

**Affiliations:** 1 School of Electronic Engineering, Beijing University of Posts and Telecommunications, Beijing 100876, China; dengzhl@bupt.edu.cn (Z.D.); liuyx@bupt.edu.cn (Y.L.); owen.hu@bupt.edu.cn (E.H.); 2 School of Artificial Intelligence, Beijing University of Posts and Telecommunications, Beijing 100876, China

**Keywords:** time frequency analysis, signal of opportunity, Nav-SOP, convolutional neural network, USRP

## Abstract

The traditional signal of opportunity (SOP) positioning system is equipped with dedicated receivers for each type of signal to ensure continuous signal perception. However, it causes a low equipment resources utilization and energy waste. With increasing SOP types, problems become more serious. This paper proposes a new signal perception unit for SOP positioning systems. By extracting the perception function from the positioning system and operating independently, the system can flexibly schedule resources and reduce waste based on the perception results. Through time-frequency joint representation, time-frequency image can be obtained which provides more information for signal recognition, and is difficult for traditional single time/frequency-domain analysis. We also designed a convolutional neural network (CNN) for signal recognition and a negative learning method to correct the overfitting to noisy data. Finally, a prototype system was built using USRP and LabVIEW for a 2.4 GHz frequency band test. The results show that the system can effectively identify Wi-Fi, Bluetooth, and ZigBee signals at the same time, and verified the effectiveness of the proposed signal perception architecture. It can be further promoted to realize SOP perception in almost full frequency domain, and improve the integration and resource utilization efficiency of the SOP positioning system.

## 1. Introduction

Global navigation satellite system (GNSS) is the most widely used navigation system. It uses satellites to broadcast positioning signals and provides positioning, navigation, and timing services for worldwide users. However, it also has some problems: (1) The signal landing power is about −130 dBm, which is easy to be interfered and spoofed. (2) The signal is easy to be blocked by obstacles, making it difficult to use in dense urban areas or indoor environments [1]. In perspective of the above-mentioned problems of GNSS, an ever-increasing amount of researchers have begun to explore reliable positioning methods that do not rely on GNSS systems.

SOP navigation utilizes all potential wireless signals in the surroundings for positioning [2]. SOP include various signals that are not specifically designed for navigation, such as digital audio broadcasting (DAB), digital video broadcasting (DVB), amplitude modulation radio (AM) and frequency modulation radio (FM), cellular signals, Bluetooth, ZigBee, Wi-Fi, and other wireless signals [3]. These signals are widespread and usually used for communication rather than navigation. We can extract useful information from SOP such as signal strength, ranging and time information for navigation. Common types of SOP are demonstrated in Table 1.

**Figure 1:**
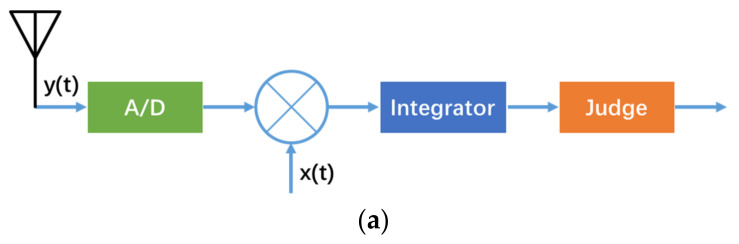

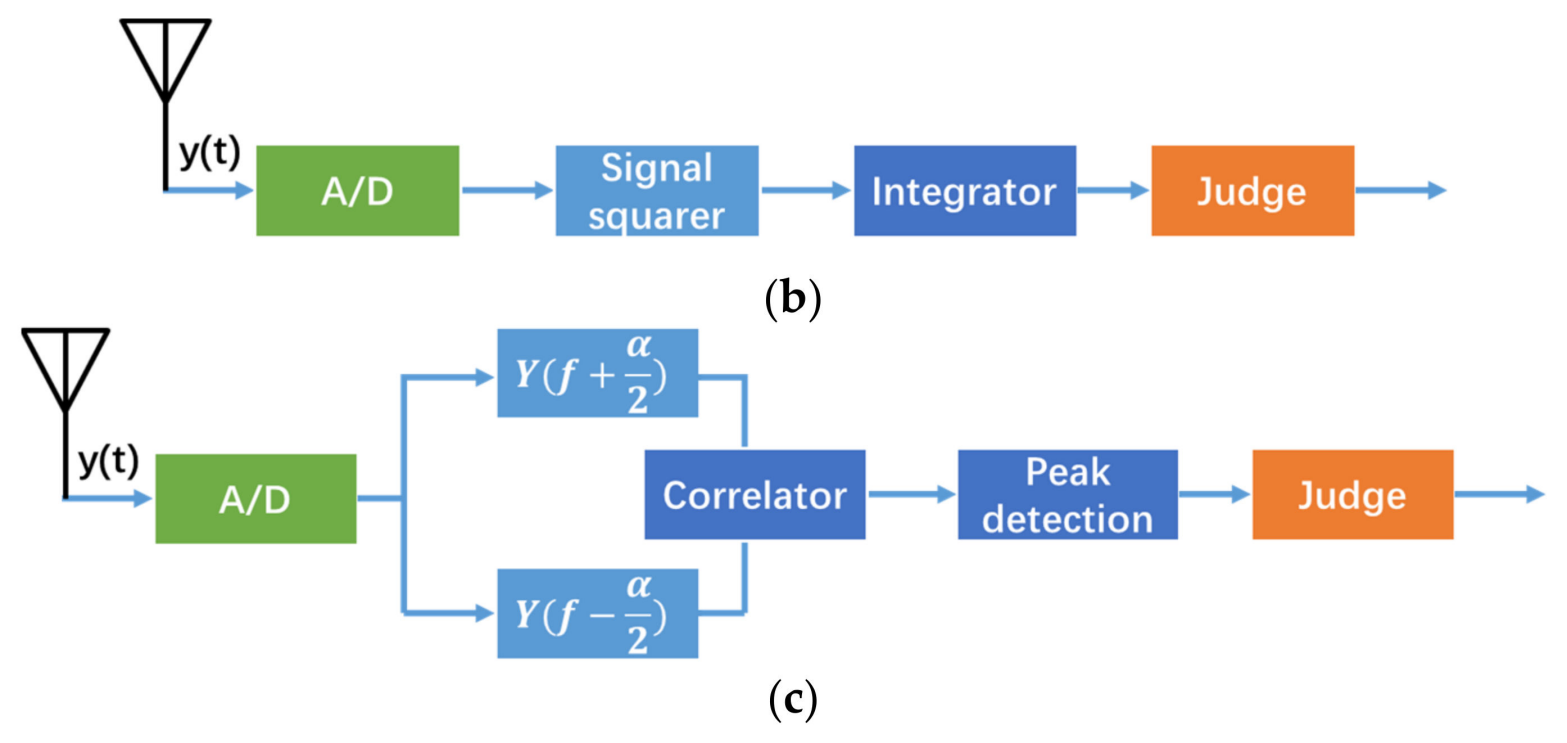
Schematic flow charts of three common detection methods. (**a**) Schematic flow chart of coherent detection; (**b**) schematic flow chart of energy detection; (**c**) schematic flow chart of cyclostationary feature detection.

The process of SOP positioning can be roughly divided into signal perception, data preprocessing, information extraction, and positioning solution [4]. Effectively identifying the SOP is the primary task of SOP navigation. It can be seen from the Table 1 that there are many types of opportunistic signals, with different distribution frequency bands, bandwidths, and signal modulation methods, which brings difficulties to signal perception. The common signal perception methods include coherent detection, energy detection, cyclostationary feature detection, etc. [5]. The schematic flow charts of three common detection methods as indicated in Figure 1.

- Coherent detection [6]:

Matched filters are a common way for coherent detection. Its advantages are high recognition efficiency and high accuracy with a short detection time, so in a sense it might be said to be an optimal detector; the disadvantage is that the relatively high computational complexity and needs prior information about the SOP, such as modulation method, modulation order, pulse waveform, data packet format, etc. It also needs time, carrier, and even channel synchronization to enable correlation with the signal, which is complicated to implement for SOP system. For different types of signals, special receivers are required too.

- Energy detection [7,8,9]:

Most signals are broadcast with a fixed frequency. By detecting the energy of the specific frequency, we can judge whether the corresponding signal exists. This method is simple to implement, has strong adaptability, and does not require more prior signal information. To measure the energy of a signal at a certain frequency, the signal output by the band-pass filter with bandwidth W is squared and integrated over the observation time. Then the calculated energy value is compared with a threshold to determine the existence of signal. The energy detection algorithm has low complexity, but the threshold is easily affected by changes in noise power and becomes invalid. Meanwhile this algorithm is not suitable for direct sequence spread spectrum (DSSS) signal, frequency hopping signals, and co-band signals (e.g., Industrial Scientific Medical (ISM) band).

- Cyclostationary feature detection [10,11]:

Communication signals usually include carrier frequencies, frequency hopping sequences, cyclic prefixes, etc., which make the signal statistical characteristics such as the mean value and correlation function periodic. However, noise does not have this characteristic, which can use to separate the noise from the target signal. This method has good detection performance even in the case of a low signal-to-noise ratio. The drawback of this method are higher complexity and longer detection time.

Over 1990s, Dr. Joseph Mitola proposed the concept of software radio [12], which has a reconfigurable software and hardware architecture. The device’s communication frequency, transmission power, modulation method, coding system, etc. can be adjusted through software configuration, effectively improved the openness and flexibility of the communication system. With the development and maturity of software radio technology, software radio-based architecture is also used by more scholars in the field of SOP positioning [13,14,15,16,17].

**Figure 2:**
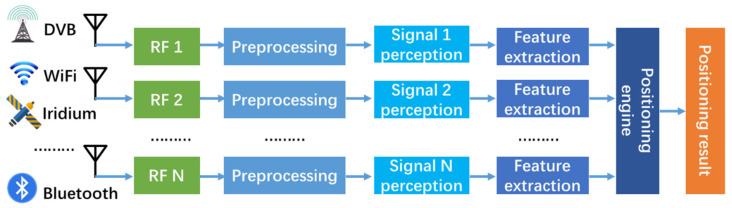
Structure diagram of traditional SOP positioning system.

At present, the usual SOP positioning system architecture is shown in the Figure 2. In order to complete signal perception and access, dedicated receivers need to equipped for each type of signal [18,19]. The equipped receiver will increase along with the types of signals, which leads to a series of problems: (1) since the lack of available signal information, all receivers need continue working to ensure the perception of all types signals, even if only no signal exist, which causes high power consumption, and hardware and energy resources waste; (2) different signals in the same frequency band (such as WiFi, ZigBee, Bluetooth, etc. in the ISM band) still need multiple devices to complete the signal perception, which does not make full utilization of hardware resources.

**Figure 3:**
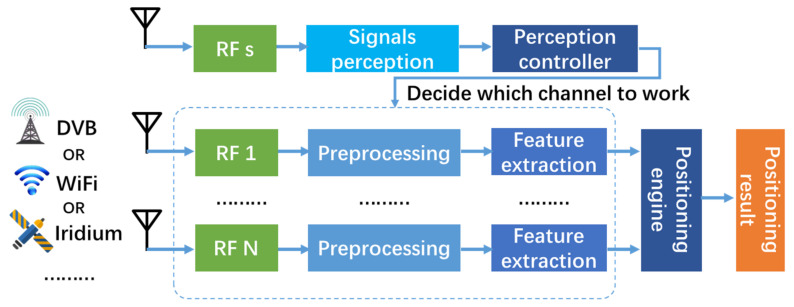
Structure diagram of a new SOP positioning system proposed by this paper.

With this as the backdrop, this paper designs a new signal perception unit of the SOP positioning system to achieve efficient SOP perception. The relationship between the perception unit and the SOP positioning system is shown in Figure 3. The task of SOP perception is completely performed by the perception unit. When the perception unit recognizes the existence of signal, it notifies the perception controller to flexibly configure the USRP equipment for target SOP and activity the corresponding receiver to start signal access. Otherwise, the receivers are in a standby state to decrease useless energy consumption. The flexibly configuration of USRP means each one can be used for all types of SOP, so we can develop a SOP positioning system with less USRP than fixed configuration system in Figure 2. It improves the system integration and resource utilization efficiency. The remaining part is to completes signal capture, tracking and demodulation and produce pseudorange, carrier phase observables, time synchronization and signal strength, etc. The positioning engine calculates the positioning result according to the signal information obtained.

The advantages of the new SOP perception architecture are as follows: (1) it can monitor hundreds of MHz bandwidth at the same time, which is related to the bandwidth of the USRP device (in this article, B210 can monitor 50 M bandwidth). Combined with time-sharing frequency hopping technique, signal perception can be implemented in a wider frequency band, but it will reduce the real-time performance of perception; (2) it can identify multiple types of signals in the same frequency band at one time. A typical example of this situation is the ISM frequency band; (3) there is no need for multiple receivers, which can save hardware resources and reduce energy consumption. The new SOP perception architecture can be extended to other SOP and realize almost all frequency domain SOP perception.

In previous work [20], we tried short-time Fourier transform (STFT) to convert signal samples into time-frequency images, speed up robust features (SURF) algorithm for feature extraction, K-means algorithm for clustering, and support vector machines (SVM) for signal classification. A simulation experiment was carried in the 2.4 GHz ISM frequency band, with Wi-Fi, Bluetooth and ZigBee as the target signals. The signal generation and perception were carried with Simulink and Matlab. The simulation experiment preliminarily verified the possibility of using time-frequency image for SOP perception. However, there are several problems in the previous work: (1) if there are multiple signals in the same time-frequency image, the result will be classified as the most likely one, and the SVM cannot identify all signal types; (2) the results are only verified by simulation, without considering the hardware implementation feasibility (which has not been actually tested).

In response to the above problems, this paper proposed an improved CNN feature extraction and classification method, built a prototype hardware system, and conducted actual experimental tests to verify the effectiveness of the designed perception architecture and algorithm. We still select Bluetooth, Wi-Fi, and ZigBee in the 2.4 GHz ISM frequency band to verify the perception ability. We also improved the time-frequency representation methods. The remaining chapters of this article are as follows: Section 2 describes the model, including the target signal and the design of the signal perception unit. Section 3 analyzes four signal time-frequency joint representation methods, and Section 4 proposes a CNN-based SOP recognition method, this section also illustrate network design, negative learning-positive learning (PL-NL) combined training process and classification result. Section 5 introduces the experimental system and experimental verification. Section 6 summarizes the work of this paper, significance for SOP positioning system, points out the shortcomings and the direction of future work.

## 2. Signal and System

### 2.1. Signal Introduction

This section briefly introduces the target signals (Bluetooth, Wi-Fi, ZigBee) in the paper and includes basic information such as channel parameters, transmission power, and access method.

- Bluetooth [21]

**Figure 4:**
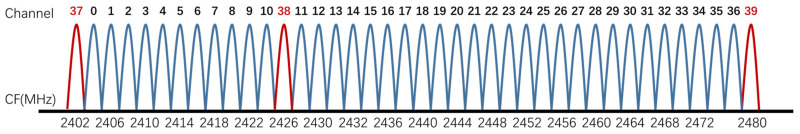
Bluetooth channel distribution.

Bluetooth is a low-power communication technology, generally used for short-distance wireless communications. The transmission power can generally be divided into three classes, namely 100 mW (class 1), 2.5 mW (class 2), and 1 mW (class 3). The modulation method of Bluetooth is Gaussian Frequency Shift Keying (GFSK), the transmission rate can reach 1 Mb/s. Bluetooth can transmit on 40 channels in the range of 2402–2480 MHz, the width of each channel is 2 MHz. When communicate with other nodes, frequency-hopping spread spectrum (FHSS) is used on 40 channels. In a non-connected broadcast mode, Bluetooth signals are broadcast on three fixed channels 37, 38, and 39. The receiver can identify the signal source by access code in broadcast data packets. The Bluetooth channel distribution is shown in the Figure 4.

- WiFi [22]

**Figure 5:**
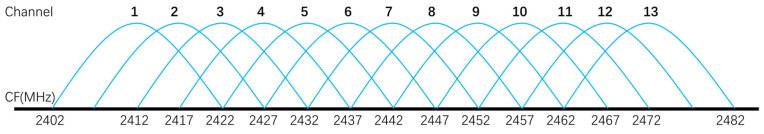
WiFi channel distribution.

The IEEE 802.11b/g/n protocol, commonly known as WiFi uses two modulation schemes. Direct sequence spread spectrum (DSSS) is used for lower bit rates transmission, and orthogonal frequency division multiplexing (OFDM) is used for higher bit rates transmission. There are 13 channels defined in the 2.4 GHz ISM frequency band (different regions may vary), the width of each channel is 20/22 MHz. The total frequency band width is 83.5 MHz so there is overlap between the channels, as shown in Figure 5. The maximum allowable power is 10 mW/MHz. The wireless access point (AP) periodically sends broadcast frames about 10 times per second to inform the existence of the WiFi network, which contains Service Set Identifier (SSID) information.

- ZigBee [23]

**Figure 6:**
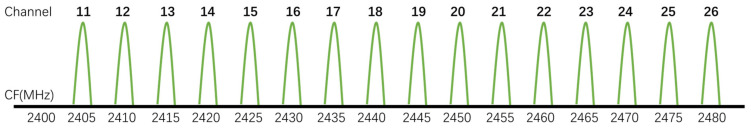
ZigBee channel distribution.

ZigBee is a low-rate wireless network, following the IEEE 802.15.4, using DSSS modulation. A total of 16 channels are defined in 2.4 GHz band and do not overlap; each channel has 2 MHz bandwidth with a 3 MHz guard interval, which is shown in Figure 6. The communication range is about 10 m, transmission rate can reach 250 kb/s. When a new ZigBee network is established, the energy detection function is used to determine the operating channel. However, dynamic channel selection is not supported.

**Figure 7:**
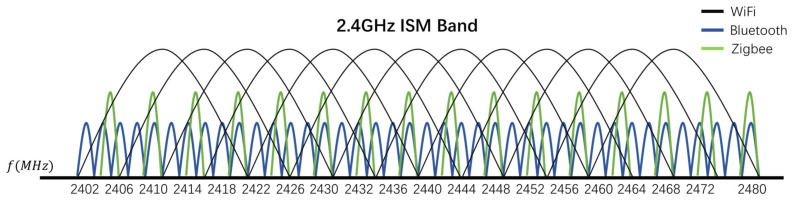
Bluetooth, WiFi, and ZigBee signal distribution in 2.4 GHz ISM frequency band.

The Figure 7 shows the relationship between Bluetooth, WiFi, ZigBee on the 2.4 GHz ISM frequency band. It can be seen that they completely overlap in the frequency domain [24].

### 2.2. System Structure

**Figure 8:**
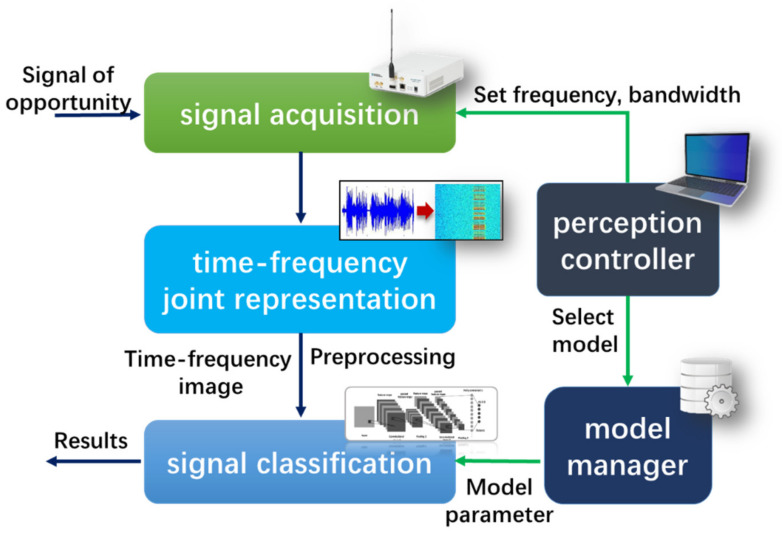
The structure of the signal perception unit.

The structure of the signal perception unit contains signal acquisition, time-frequency joint representation, perception controller, model manager and signal classification. The relationship of each part is shown in Figure 8.

- Perception controller

**Figure 9:**
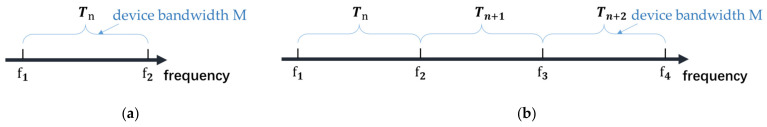
Perception strategy: (**a**) fixed frequency mode; (**b**) frequency hopping mode.

The perception controller is the control center of the perception unit, which completes the configuration according to different needs. Its perception strategy contains fixed frequency mode and frequency hopping mode. The frequency hopping mode is design to solve the contradiction between the limitation of hardware resources (such as the bandwidth of the USRP, the transmission rate of the interface) and the wider target frequency band. In this mode, according to the monitoring frequency range $f_{1},f_{2}$, and the device bandwidth M, the monitoring frequency domain can be segmented into pieces. The controller changes the center frequency periodically to realizes the monitoring of a wider frequency band, but the frequency hopping causes the perception hysteresis. The schematic diagrams of the two modes shown in Figure 9.

- Signal acquisition

After the perception controller completes the configuration of the hardware parameters (center frequency, sampling rate, etc.), USRP starts the signal acquisition whose process includes mixing, AD sampling, data buffering, etc. The obtained data will be transferred to LabVIEW software on PC via UHD driver and USB 3.0, waiting for further processing.

- Time-frequency joint representation

In order to obtain more abundant signal characteristics, this paper uses time-frequency joint representation to convert 1D time-domain signals into 2D time-frequency images, which present the time-frequency joint characteristics of the signal. Each pixel in image represents the energy of the wireless signal at a certain frequency and time. The entire image shows the wireless signal energy distribution over the frequency domain and a period of time. Specifically, the transform is implemented based on the LabVIEW software.

- Preprocessing

The acquired time-frequency images need further processed for signal classification or data set, including grayscale, size normalization, etc.

- Signal classification

This part classifies time-frequency image through a pre-trained CNN model. In order to adopt different frequency bands or types of signals, the model can be updated according to the parameters given by the model manager. This paper implements an improved CNN classification method through python and pytorch library.

- Model manager

The model manager stores multiple sets of CNN parameters, different parameters correspond to different frequency bands and signal types. The model manager selects appropriate parameters according to the instructions of the perception controller and send them to the signal classification part to complete the model update.

## 3. Time–Frequency Representation

**Figure 10:**
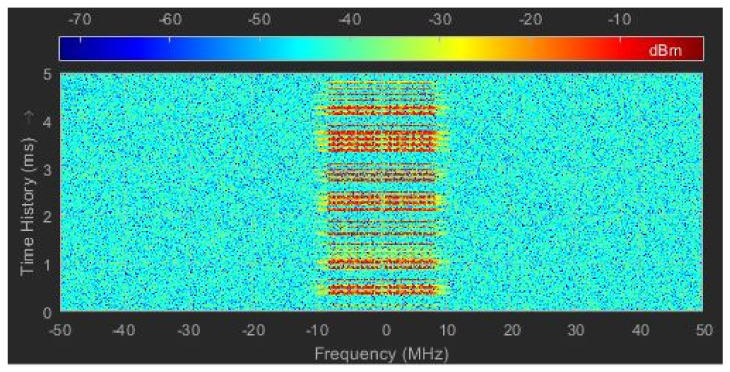
Time−frequency image.

Signal analysis can be carried out in the time or frequency domain by Fourier transform or inverse transform. However, the Fourier transform is a kind of overall transform, which is only suitable for stationary and deterministic signals, and cannot reflect the changes of signal frequency characteristics over time. To analyze the time-varying frequency information of a signal, time-frequency representation (TFR) is needed. TFR transforms the signal from single time/frequency domain into a time-frequency 2D feature image [25], which reflects the time-frequency joint characteristics of the signal. A WiFi time-frequency image is shown in Figure 10.

Since the non-parametric time-frequency analysis method does not require prior knowledge of the signal, the time and frequency resolution obtained does not depend on the specific signal, and is more suitable for the scenario of SOP perception. Commonly used non-parametric time-frequency analysis contains linear and nonlinear methods [26,27]. Typical linear analysis includes STFT, Continuous wavelet transform (CWT), etc., and typical nonlinear analysis includes Wigner-Ville distribution (WVD), Cohen Classes, etc.

### 3.1. Short-Time Fourier Transform

The basic idea of STFT is to use a window function for signal interception, and assume that the signal is stable within the window. Fourier transform is used to analyze the intercepted signal, and then move the window function along the signal time direction to obtain the time-frequency distribution relationship. The STFT of signal *x*(*t*) is expressed as:

(1) $$STFT\left(\tau ,\;f\right)=\int_{-\infty }^{\infty }x\left(t\right)g\left(t-\tau \right)e^{-j2\pi ft}dt$$

where *x*(*t*) is the target signal and *g*(*t*) is the window function [28].

In the process of STFT, the length of the window determines the time and frequency resolution of the time-frequency image. The longer the window length, the higher the frequency resolution after Fourier transform and the worse the time resolution. The length of the window needs to be adjusted according to the specific situation.

### 3.2. Continuous Wavelet Transform

The continuous wavelet transform of the signal *x*(*t*) is expressed as:

(2) $$CWT\left(a,b\right)=\frac{1}{\sqrt{a}}\int_{-\infty }^{\infty }x\left(t\right)\bar{w}\left(\frac{t-b}{a}\right)dt$$

where $\bar{w}$ is the complex conjugate of *w*, *w* is the mother wavelet function that satisfies the admissible condition, *a* is the expansion factor, and *b* is the translation factor [29]. The commonly used mother wavelet function is Morlet wavelet, and its expression is:

(3) $$w\left(t\right)=\pi^{-\frac{1}{4}}e^{j2{\pi f}_{0}t}e^{-\frac{t^{2}}{2}}$$

### 3.3. Wigner-Ville Distribution

The WVD is a basic non-linear analysis method, which was originally proposed by Wigner in quantum mechanics, and the Wigner-Ville distribution of signal *x*(*t*) is expressed as:

(4) $$WVD\;\left(t,\;f\right)=\int_{-\infty }^{\infty }z\left(t+\frac{\tau }{2}\right)\bar{z}\left(t+\frac{\tau }{2}\right)e^{-j2\pi ft}d\tau \;z\left(t\right)=x\left(t\right)+j\int_{-\infty }^{\infty }\frac{x\left(u\right)}{t-u}du=x\left(t\right)+jH\left[x\left(t\right)\right]$$

where *z*(*t*) is the analytical signal of *x*(*t*), *H*[*x*(*t*)] represents the Hilbert transform of signal *x*(*t*), and $\bar{z}$ is the complex conjugate of *z* [30].

If $x\;\left(t\right)=x_{1}\left(t\right)+x_{2}\left(t\right)$, then:

(5) $$WVD\;\left(t,\;f\right)={WVD}_{x1}\left(t,\;f\right)+{WVD}_{x2}\left(t,\;f\right)+2Re\left[{WVD}_{x1x2}\left(t,\;f\right)\right]$$

where ${WVD}_{x1x2}\left(t,\;f\right)$ is the cross term of the Wigner-Ville nonlinear distribution:

(6) $${WVD}_{x1x2}\;\left(t,\;f\right)=\int_{-\infty }^{\infty }x_{1}\left(t+\frac{\tau }{2}\right)\bar{x_{2}}\left(t+\frac{\tau }{2}\right)e^{-j2\pi ft}d\tau$$

### 3.4. Cohen Classes

The Cohen classes time-frequency analysis is a modification of the WVD, which can be expressed in a unified form:

(7) $$CTFD\left(t,f\right)=\iint \phi \left(\tau ,\;\theta \right)\mathrm{WVD}\left(t-\tau ,f-\theta \right)\;d\tau d$$

In the formula, WVD is the Winger-Ville distribution, and *ϕ* (*τ*, *θ*) is called the kernel function [31].

Commonly used Cohen Classes distributions include pseudo-Wigner-Ville distribution (PWD), smoothed Wigner-Ville distribution (SWD), Born-Jordan distribution (BJD), Generalized rectangular distribution (GRD), Choi-Williams distribution (CWD), Zhao-Atlas-Marks distribution (ZAMD), etc.

### 3.5. Effect Analysis

**Figure 11:**
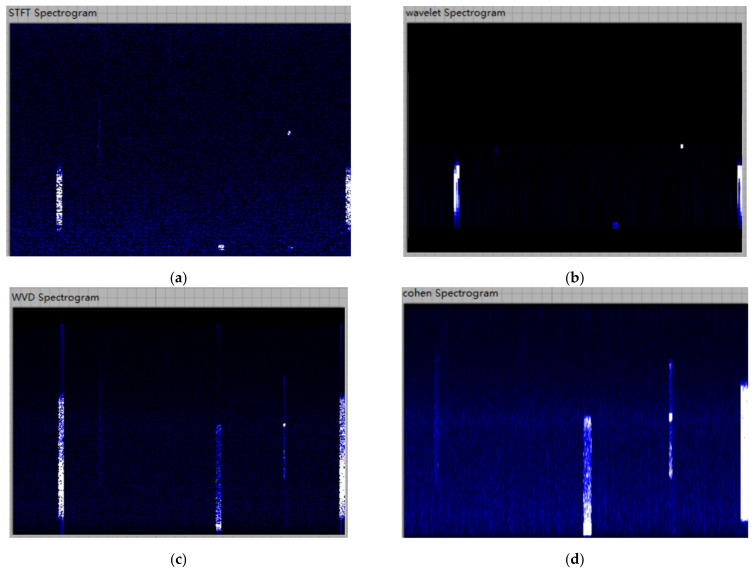
Comparison of four types time-frequency images: (**a**) STFT; (**b**) CWT; (**c**) WVD; and (**d**) Cohen.

We select a same segment of signal for experiment to compare the four time-frequency analysis methods, and the results are shown in the Figure 11. The abscissa of image represents time, and the ordinate represents frequency. The lighter the color of the pixel, the higher the power. It can be seen that the time-frequency distribution of Cohen and WVD has obvious cross-term interference. Between STFT and CWT, the CWT’s signal energy more concentrated. Therefore, we select CWT for time-frequency representation in this article.

Through time-frequency joint representation, we have completed the transformation of signal information from time/frequency-domain to time-frequency joint characteristics, providing more usable information for signal perception. The next step is to send the obtained time-frequency image to the CNN for model training and signal perception.

## 4. CNN-Based Signal Classification Model

Machine learning is commonly used to instead artificial visual interpretation in image classification which can be roughly divided into: shallow learning and deep learning [32]. Shallow learning includes: SVM, Boosting, Logistic Regression, etc. Deep learning includes: convolutional neural network (CNN), recurrent neural network (RNN), generative adversarial network (GAN), etc. A large number of experiments and practices have verified that the shallow learning performs poorly in processing high-dimensional data, but deep learning makes up for this shortcoming. By using multi-level non-linear processing units, it has advantages in extracting deep structural features, and is more suitable for tasks such as visual recognition [33,34], audio recognition [35,36], and natural language processing [37,38].

As a deep feedforward network, CNN’s core is to simulate the learning behavior of the human brain by constructing a neural network model, and optimize the parameters of the CNN model through training iterations [39,40]. The classic CNN models contains LeNet-5, AlexNet, ZF-Net, VGGNet, GoogLeNet, ResNet, and DenseNet.

AlexNet [41] is a CNN framework proposed by Alex and Hinton when they participated in the 2012 ImageNet competition. They introduce the Relu activation function and Dropout to improve training speed and prevent overfitting. The advantages of AlexNet are simple calculation and fast convergence speed.

### 4.1. CNN Structure Design

The CNN consists of convolutional, pooling, and fully connected layers. The theoretical basis of the convolutional layer is the concept of receptive fields in biology, which can greatly reduce the parameters required for neural network training. Pooling, also known as down-sampling, is used to reduce the amount of data while retaining useful information. By superimposing the convolutional layer and the pooling layer, it forms one or more fully connected layers to achieve higher-order reasoning capabilities.

**Figure 12:**
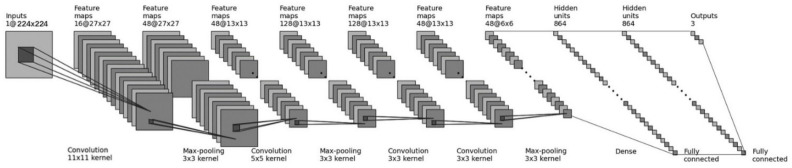
CNN network structure designed in this paper.

In this paper, a CNN model is designed based on the Alexnet architecture for signal classification and is streamlined to reduce the requirements for device performance. The network structure is in Figure 12:

It contains four pairs of convolutional layers and pooling layers (C1-P1, C2-P2, C3-P3, and C4-P4), followed by two fully connected layers (FC1 and FC2) and an output layer (FC3). The main purpose of the convolutional layer is the feature abstraction and extraction, while the pooling layer is responsible for feature fusion and dimensionality reduction. The fully connected layer is responsible for logical inference, in which the first one is used to link the output of the convolutional layer, remove the spatial information (number of channels), and turn the three-dimensional matrix into vector. Each convolutional and fully connected layers’ output, except the last output layer, are connected to rectified linear unit (ReLU), which helps to alleviate the gradient disappearance or explosion, and speed up the training process.

After analysis, the color of the time-frequency image is of little significance to signal classification, meanwhile the more important things are signal pattern character and spatial distribution. Therefore, during preprocessing the time-frequency image obtained is directly transformed into a 224 × 224 grayscale image, so the input size of the network is 224 × 224 pixels. The convolution kernel size of the first convolution layer is 11 × 11 × 16, stride = 4 and padding = 2. The total parameters number of this layer is (11 × 11) × 16 = 1936, which represents the weight of the layer. The output size of each convolution kernel in the first layer is (224 − 11)/2 + 1 = 55, and the output size of the C1 is 55 × 55 × 16. The second layer is a pooling layer (P1), with a size of 3 × 3 and stride = 2. The output size of the kernel is (55 − 3/2 + 1) = 27, so the output size of this layer is 27 × 27 × 16. All parameters of the pooling layer are hyper-parameters and do not need to be learned. Similarly, we can calculate the size of each convolution and pooling layer. In the end there are 2 fully connected layers with 864 neurons in each layer, whose parameters are fully connected weight coefficients. We use the dropout layer after the fully connected layer to avoid overfitting.

Since the existence of signal is independent for each other, this is a multi-label classification problem. So, we replaced the original softmax with Sigmoid function in the last layer (FC3). The output probability of each signal is between [0, 1]. If the output is greater than the probability threshold (usually 0.5), we considered that corresponding signal exists.

### 4.2. Data Collection

**Figure 13:**
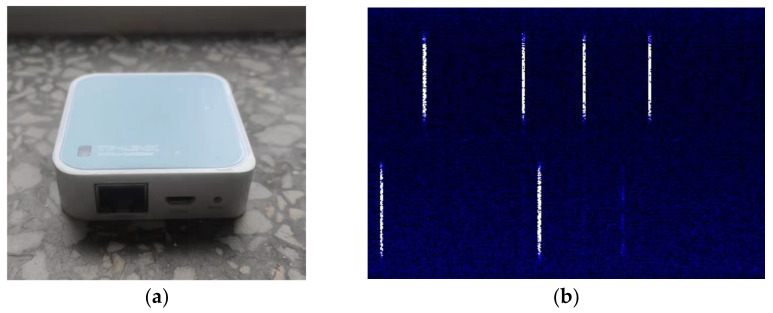
WiFi equipment: (**a**) photo of TL-WR802N; (**b**) WiFi signal time-frequency image.

Before the network training, a data set must be collected first for model training and training effect evaluation. This article uses hardware equipment to generate signals for testing and data acquisition. The equipment selection is as follows:

We select TP-Link mini wireless router node TL-WR802N as WiFi equipment, which main control chip is Qualcomm QCA9533. It follows IEEE802.11n standard, and runs in AP mode by default, transmission power <20 dbm. The photos of the TL-WR802N and the time-frequency image are in Figure 13.

**Figure 14:**
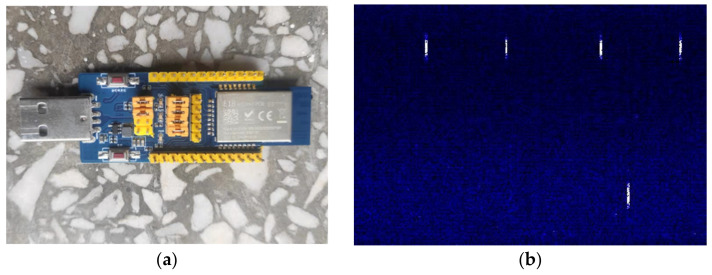
ZigBee equipment: (**a**) photo of E18-TBL-01; (**b**) ZigBee signal time-frequency image.

We select the E18-TBL-01 module produced by EBYTE as ZigBee equipment. The main control chip of the module is TI’s CC2530 chip, which integrates an enhanced 8051 CPU, follows the IEEE802.15.4 standard. Transmit power can set as 4.5/20/27 dBm. The module works in broadcast mode by default. The photos of the E18-TBL-01 and the time-frequency image are in Figure 14.

**Figure 15:**
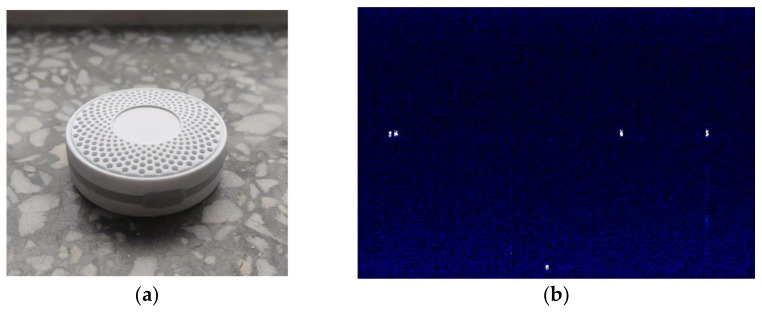
Bluetooth equipment: (**a**) photo of iBeacon node; (**b**) Bluetooth signal time-frequency image.

The Bluetooth equipment uses Social Retail’s iBeacon node, and the main control chip is TI’s CC2541 Bluetooth chip. The iBeacon carries on BLE broadcasting whose frame period is 500 ms, and transmission power is 0 dBm. The photos of the Bluetooth iBeacon and the time-frequency image are in Figure 15.

**Figure 16:**
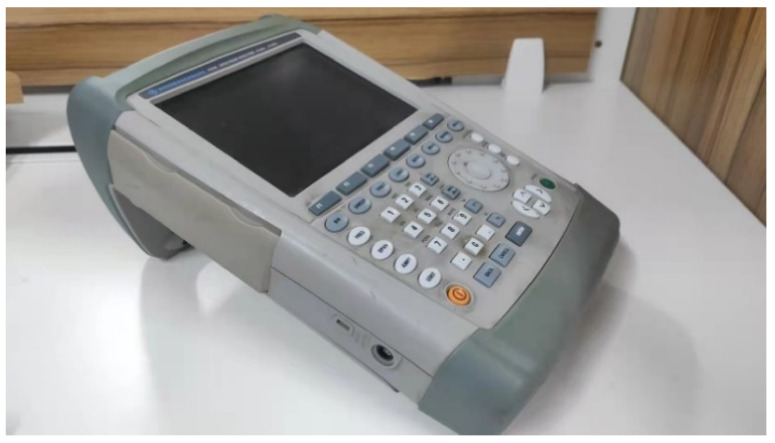
Rohde and Schwarz FSH8 Spectrum Analyzer.

**Figure 17:**
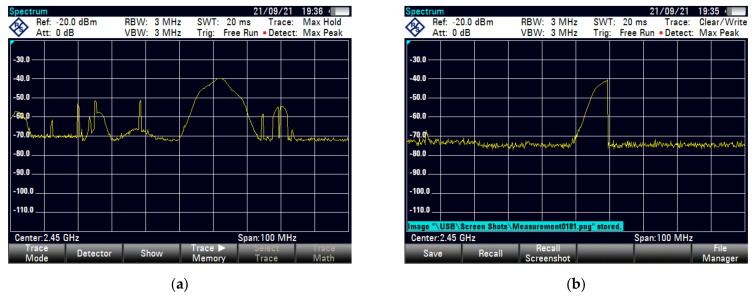
Interference signal detection result by spectrum analyzer: (**a**) max hold model detection result; (**b**) clear/write model detection result.

We choose a spacious environment for signal acquisition and to ensure that there was no interference signals. In order to monitor possible external interference sources at the test area (such as other Wi-Fi equipment), we used Rohde & Schwarz’s FSH8 spectrum analyzer which is shown in Figure 16. Figure 17 shows the spectrum analyzer detection result measured in two ways: (a) using the max hold mode to measure the maximum level within a period of time; and (b) using the clear/write mode, observe whether there is a jump on the 2.4 G spectrum. Perform interference detection before each experiment to check external interference, so as to avoid results bias.

**Figure 18:**
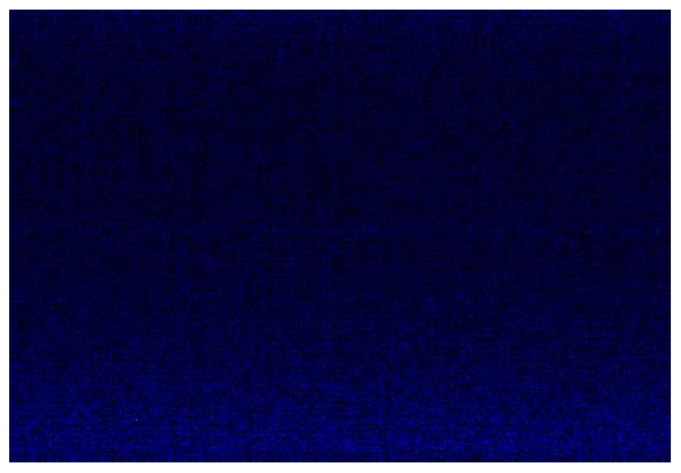
The time-frequency image of noise signal.

If we acquire signal time-frequency images at the same time, the acquired image should only contain low-power noise signal and USRP device thermal noise which is shown in Figure 18.

**Figure 19:**
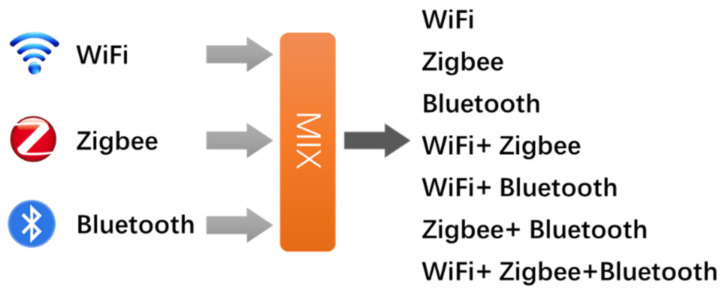
Types of signal combinations.

After confirming no external interference sources in the experimental environment, place the signal source equipment and turn on the signal acquisition system to collect time-frequency images under different signal combinations. The number of each type of node in the working state is variable, and should include all the signal combinations which is better in conformity with practical channel environment. Three types of signals can enumerate seven types of signal combination situations, as shown in Figure 19. If the number of working signal source can change at the same time, the combination will be more complicated, so we do not list them one by one here.

**Figure 20:**
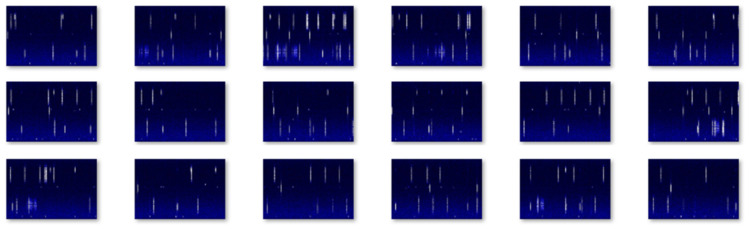
Part of the data set.

We chose all situations in the Figure 19 above as data set labels, and each label collected at least 200 pictures. In the actual acquisition process, images with weak signal characteristics or no signal at all will appear, and these poor-quality data need to be manually eliminated. Finally, we use 80% of the data set as the training set, 20% as the validation set, and collect other 20 images for each type of label as the test set. Figure 20 shows a part of the data set.

### 4.3. Model Training

**Figure 21:**
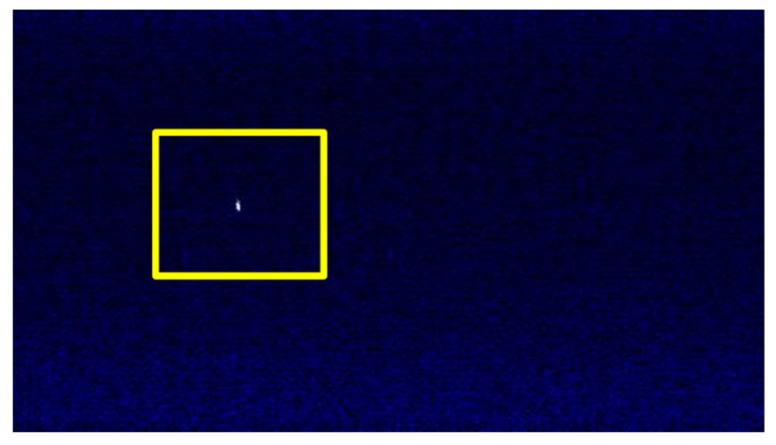
Bluetooth time-frequency image with unobvious characteristics.

Sometimes the signal pattern in the time-frequency image is small and sparse, the features are not obvious. This often happens when the target signal has long broadcast cycle, weak power and small bandwidth (for example, Bluetooth). An example is shown in the Figure 21, the white dot in the area enclosed by the yellow box in the figure are Bluetooth signal pattern.

**Figure 22:**
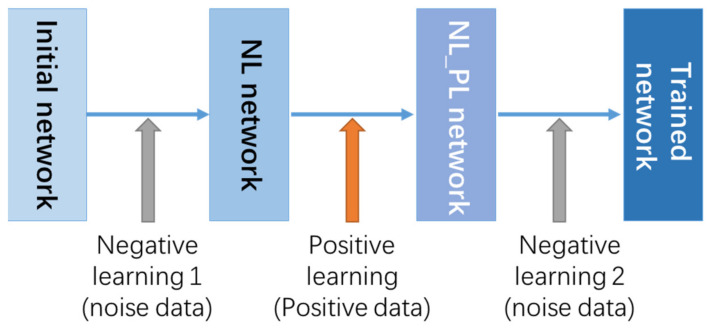
The training process combined NL and PL.

This will cause the model to learn the noise features incorrectly during the training process and cause over-fitting. So, we introduce a negative learning (NL) training method to prevent CNN from overfitting noisy data which is proved by Kim [41]. NL method does not require any prior knowledge of noise data such as type and quantity. Different from the positive label data used in positive learning (PL) which contains the target feature information that the model focuses on, the negative label data can tell the model about the feature information of noise and interference information, which helps to distinguish the useless features. By combining PL and NL, we can improve accuracy while ensuring training speed. PL can quickly reduce the loss, but it is easy to overfit in the end. The obtained model after PL is then subjected to NL to correct the over-fitting of the noise and improve the recognition accuracy. This article uses a combination of two NL and one PL for training. The training process is shown in the Figure 22.

In order to obtain the best classification performance, we need to adjust three hyperparameters which are the initial learning rate, the mini-batch size, and the training iterations number. We set different hyperparameter values for the three training processes, and conduct a series of training to try different parameter combinations. The final parameter values are shown in Table 2.

### 4.4. Training Result

**Figure 23:**
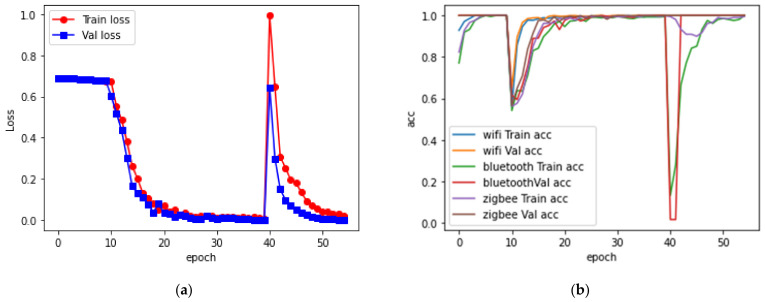
Training curves: (**a**) loss curve; (**b**) accuracy curve.

In a total of 55 iterations of training process, the loss function and training accuracy curve are shown in the Figure 23. It can be seen that after the first 40 iterations of training, the loss function curve gradually decreased to a lower level. However, in the 41st training process, both the loss curve and the accuracy curve showed great changes which means the model has been overfitted. The second negative learning completed the correction of the over-fitting, and the loss function and accuracy curve returned to a normal level. The test results of the finally trained model on the test set are shown in the Table 3.

## 5. Experiments and Performance Evaluation

### 5.1. Perception Experiment

#### 5.1.1. Experimental System

**Figure 24:**
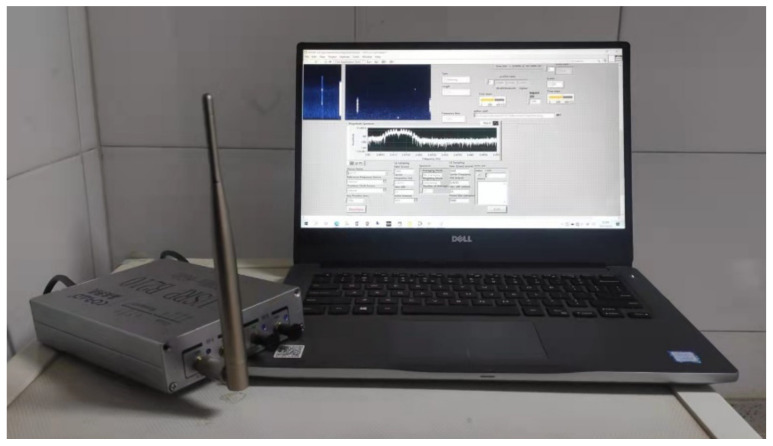
SOP perception system.

We built a SOP perception system based on the USRP platform. The hardware uses B210 USRP produced by Ettus and a DELL notebook. The device connection relationship is shown in the Figure 24, USRP’s radio port is connected to a standard 2.4 GHz omnidirectional antenna, and data port is connected to the laptop through the USB3.0 interface.

USRP B210 integrates a AD9361RFIC direct conversion transceiver, providing up to 56 MHz real-time bandwidth, and the radio frequency range is from 70 MHz to 6 GHz. The onboard signal processing and control of the AD9361 is performed by a Spartan6 XC6SLX150 FPGA, which is connected to the host PC using USB3.0, and the PC performs further processing on the collected data.

The notebook model is DELL’s P74G, with i7-8550U dual-core CPU, 8 GB RAM, Windows 10 operating system, the installed software includes USRP Universal Hardware Driver (UHD) driver, LabVIEW 2020, python 3.8. Remove the internal wireless network card during the test to avoid interference.

**Figure 25:**
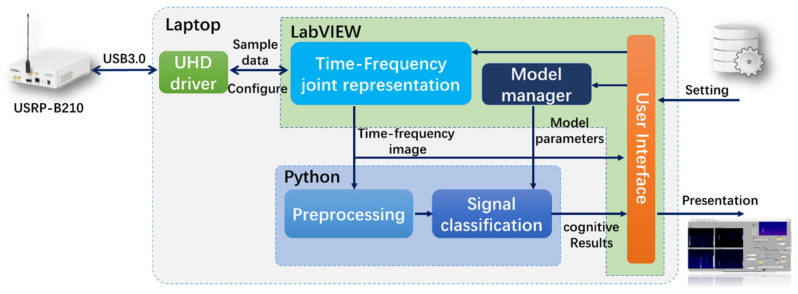
The structure and data flow of the SOP perception system.

The structure and data flow of the entire perception system is shown in the Figure 25.

**Figure 26:**
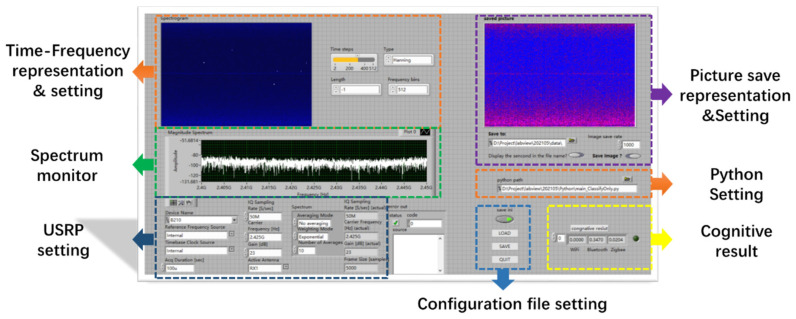
System software interface.

The USRP completes the sampling of the wireless signal and sends the data to the PC via UHD and USB3.0. LabVIEW software completes the subsequent signal processing and interactive interface. Signal processing includes time-frequency image representation, image preprocessing, perception model management, signal classification, etc. The signal classification uses the python node to run the pre-trained CNN model through LabVIEW. We use python to implement the CNN model based on the pytorch library. The system software interface is shown in Figure 26. The functions include system settings, spectrum monitoring, real-time preview, classification results display, data collection and storage, etc.

#### 5.1.2. Experimental Scenarios

**Figure 27:**
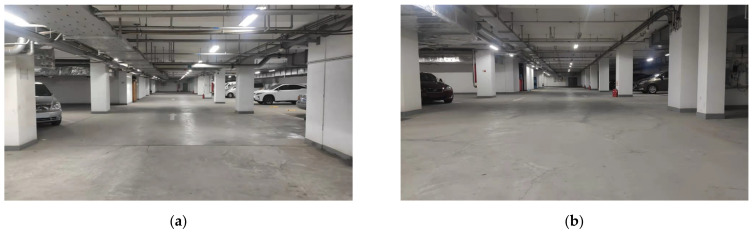
Experimental environments: (**a**) B1 of parking lot; (**b**) B2 of parking lot.

In order to test the SOP perception effect, we deployed multiple WiFi, Bluetooth, ZigBee signal nodes in the actual scene, and used the built perception system for signal perception testing. The experiment was chosen to be carried out in a two-story underground parking lot where has no 2.4G wireless equipment. Figure 27 shows a real view of the test site.

**Figure 28:**
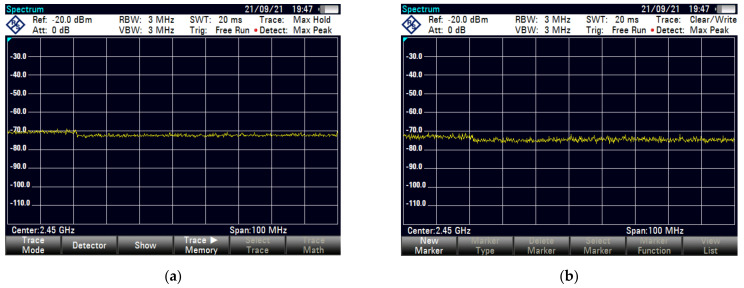
Spectrum analyzer detection results show no interference source: (**a**) max hold model; (**b**) clear/write model.

In advance, we make sure that there was no external interference in the 2.4 GHz frequency band in the test site by using an Agilent spectrum analyzer. The test results are shown in the Figure 28.

**Figure 29:**
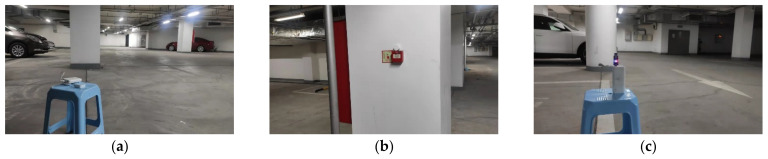
The deployed signal nodes: (**a**) Wi-Fi node; (**b**) Bluetooth node; and (**c**) ZigBee node.

After external interference check, we arranged the signal nodes in different areas. The layout need consider the influence to perception with different numbers and combinations of signal sources. The deployed nodes are shown in Figure 29.

**Figure 30:**
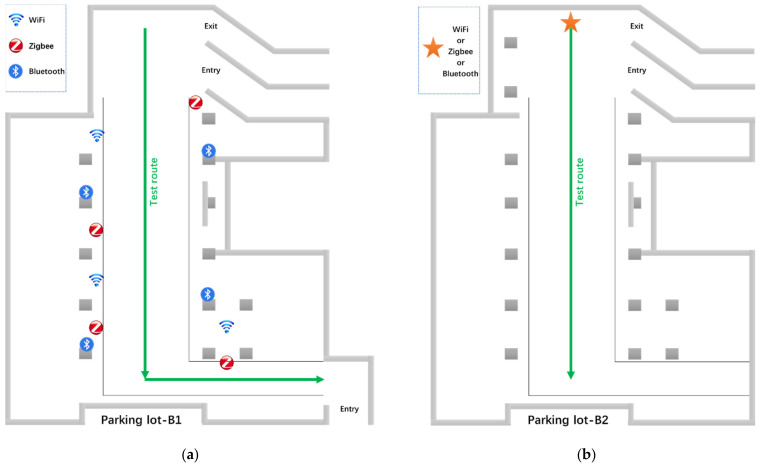
Test site plan and signal source layout location: (**a**) B1 of parking lot; (**b**) B2 of parking lot.

On the B1 layer, we deploy multiple types of signal nodes at the same time to test the system’s signal perception ability in a complex wireless environment where multiple signals coexist. On the B2 layer, we only deploy one type of signal at a time to test the system’s ability to percept specific signal at different distance. The floor plan and signal source layout position are shown in Figure 30. The green line represents the walking route of tester.

#### 5.1.3. Experimental Result

**Figure 31:**
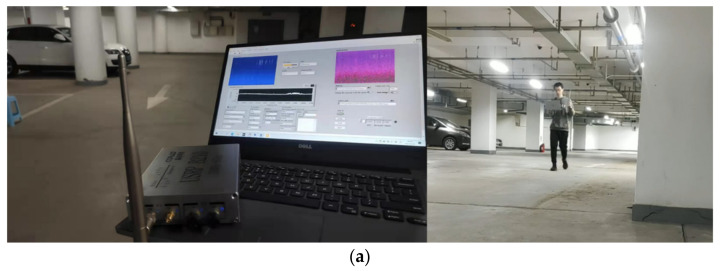

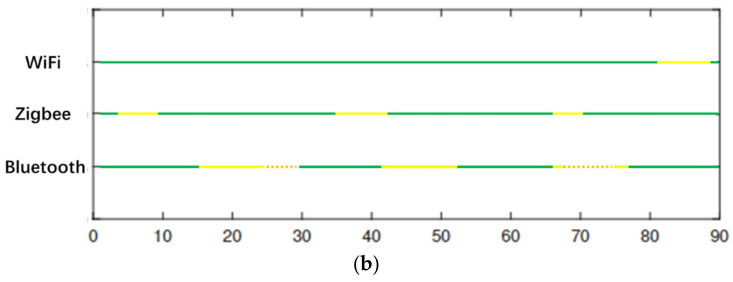
Experiment and results in B1: (**a**) experimenting in parking lot; (**b**) perception result in parking lot B1.

In the B1 the tester holds the perception system and passes through the test area according to the route. The perception results are shown in Figure 31. We use line graphs to indicate the signal perception result. The dashed line indicates that the signal is not recognized at all, and the solid line indicates that the signal is recognized. At the same time, the red solid line represents the perception result is error, yellow solid line represents the signal perception result is correct but unstable, and green represents the result is correct, stable, and continuous.

Experimental results show that the system can simultaneously perceive WiFi, Bluetooth and ZigBee signals in a mixed wireless signal environment. However, accidental misrecognition and unstable recognition also occurred during the experiment. The reason of unstable recognition or even unrecognizable during the experiment are as follows: (1) the weak signal power causes weak signal characteristics. When the signal power is less than the noise, signal perception failed; (2) the signal broadcast cycle is too long to guarantee that the signal will be captured in each time-frequency image. These two situations are more common in the Bluetooth signal perception. Due to its low power consumption design, the signal transmission power is lower and the period is longer. The weak signal power also easy to appear due to ‘non-line of sight’.

**Figure 32:**
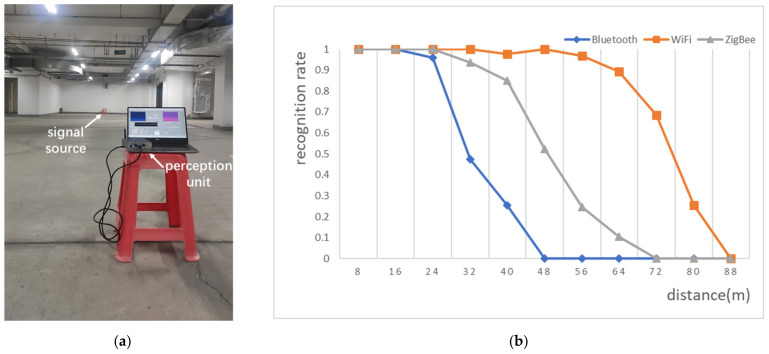
Experiment and results in B2: (**a**) experimenting in parking lot; (**b**) the recognition rate at different distances.

In the B2, we tested the perception ability at different distances, respectively. The distance between signal source and test points are a multiple of 8 m. At each test point, we continuously record the perception results for 2 min and calculate the recognition rate. The result is shown in Figure 32.

It can be seen from the experimental results that the effective distance of perception is: WiFi > ZigBee > Bluetooth, and the main factors that affect the perception are signal power and signal bandwidth. The stronger the power, the wider the signal bandwidth, and the more obvious the characteristics of the target signal on the time-frequency image, the easier it is to be accurately identified, and the longer the perception distance. It should be noted that when the signal received power is less than the noise, no signal characteristics can be reflected in the time-frequency image, and the perception method is invalid.

### 5.2. Energy Efficiency Evaluation

After confirming the effectiveness of the perception architecture proposed in this article, we can further evaluate its improvement in energy efficiency. As mentioned above, the sensing result of the SOP perception unit can not only help the flexible configuration of the USRP device, but also determine whether it enters the standby state to reduce power consumption. Assume that the access USRP device’s running power consumption is $P_{1}$, and $P_{1}^{\prime }$ in standby mode. The perception USRP device’s running power consumption is $P_{2}$. The saved power can be calculated as $\left(P_{1}-P_{1}^{\prime }\right)\times N\prime -P_{2}$, where $N^{\prime }$ is the number of USRP switched to standby mode according to the perception result. The energy efficiency improvement percentage can be further calculated as $\frac{\left(P_{1}-P_{1}^{\prime }\right)\times N\prime -P_{2}}{P_{1}\times N}$.

**Figure 33:**
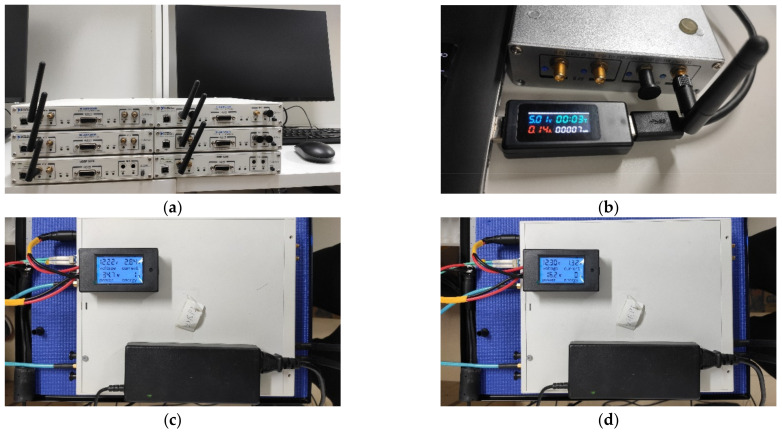
Energy efficiency evaluation experiment: (**a**) a SOP positioning system with six X310 and one B210; (**b**) P_2_: the running power of B210; (**c**) P_1_: the running power of X3100; (**d**) $P_{1}^{\prime }$: the standby power of X310.

We take a SOP positioning system composed of six X310 and one B210 as an example for power consumption evolution, where X310s are responsible for signal access and B210 is responsible for signal perception. By using a DC power meter, we measured the power consumption of the device in different states: The $P_{1}$ and $P_{1}^{\prime }$ of the X310 is about 34.7 w and 16.2 w; the $P_{2}$ of the B210 is about 0.7 w, which is shown in Figure 33.

**Figure 34:**
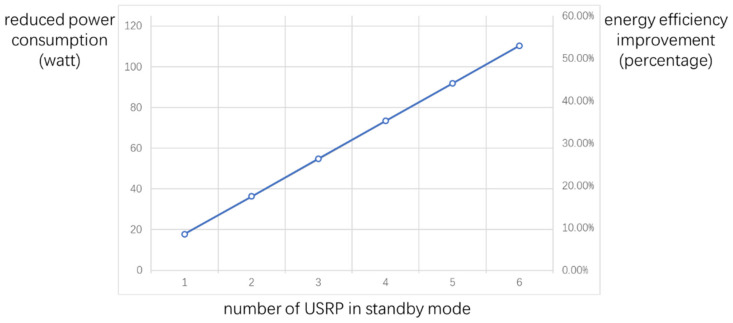
Relationships between the reduced power consumption, percentage of energy efficiency improvement, and the number of USRP switched to standby mode.

Based on these data, the reduced power consumption and percentage of energy efficiency improvement at different $N^{\prime }$ can be calculated, as shown in Figure 34. It can be seen that the power consumption can be reduced by about 10% to 50% (20 w–110 w) in this system.

## 6. Conclusions

This paper proposes a new signal perception architecture for SOP positioning system and completed the implementation. By separating the signal perception function, it reduces hardware and energy resources waste caused by multiple devices continuous working in traditional method. The core is an CNN-based SOP classification model and signal time-frequency joint representation. We use CWT to complete the signals time-frequency joint representation, and designs a CNN-based model for feature extraction and classification to time-frequency images. This paper introduces the NL-PL joint training method, which can suppress overfitting to noise data effectively. Compared with the previous work with SURF + K-means + SVM method, the recognition rate is higher (more than 97%), and solved the problem of recognize mixed signals in same time-frequency image. We also build a prototype system through USRP and LabVIEW, and verified the perception ability of 2.4 GHz ISM signals (WIFI, Bluetooth, ZigBee) in the underground parking lot. The experiment result proved the effectiveness of the design. The perception architecture proposed in this paper can be extended to other opportunistic signal and realize almost all frequency domain and all kinds SOP perception. The efficient realization of the SOP perception function can promote the further integration and upgrade of the SOP positioning system.

In response to the problems exposed in research and experiments, the subsequent research directions are as follows:

1. Introduce noise suppression methods to solve the perception failure when the target signal power is at the same level of noise, and improve the sensitivity of perception;
2. Select USRP equipment with better performance to realize wider bandwidth SOP perception;
3. Combine the perception unit proposed with SOP positioning system to carry out positioning experiments.

## Figures and Tables

**Table 1:** List of common SOP.

Signal	Frequency	Bandwidth
WiFi	2.4 GHz/5 GHz	20 MHz/40 MHz/80 MHz
Bluetooth	2.4 GHz	1 MHz
ZigBee	2.4 GHz	2 MHz
DVB-T	40–200 MHz	8 MHz
GMS	900, 1800 MHz	200 kHz
Iridium	1620 MHz	41.67 kHz

**Table 2:** Hyperparameter setting for the three training processes.

	Learning Rate	Batch Size	Training Iteration Number
Negative learning 1	0.000002	30	10
Positive learning	0.0003	30	30
Negative learning 2	0.00001	30	15

**Table 3:** Test set verification results.

	Learning Rate	Batch Size	Training Iteration Number
Negative learning 1	0.000002	30	10
Positive learning	0.0003	30	30
Negative learning 2	0.00001	30	15

## Data Availability

Data are available in a publicly accessible repository that does not issue DOIs. Publicly available datasets were analyzed in this study. These data can be found here: (https://github.com/chgmqh/SOP_perception, accessed on: 16 October 2021).
